# Supplementary material for: Proteomic Time-Course Analysis of the Filamentous Anoxygenic Phototrophic Bacterium, Chloroflexus aurantiacus, during the Transition from Respiration to Phototrophy
Source: Microorganisms. 2022 Jun 25;10(7):1288. doi: 10.3390/microorganisms10071288 (PMC9316378; doi:10.3390/microorganisms10071288)
Supplement: Supplementary file 1 [file microorganisms-10-01288-s001.zip › Chloroflexus proteome Supplementary information.pdf]

Supplementary information for

Proteomic Time-Course Analysis of the Filamentous Anoxygenic Phototrophic Bacterium, *Chloroflexus aurantiacus*, during the Transition from Respiration to Phototrophy

Authors

Shigeru Kawai[1,2,*], Shigeru Shimamura[1], Yasuhiro Shimane[1,3], Yusuke Tsukatani[1,*]

Affiliation

[1]Institute for Extra-Cutting-Edge Science and Technology Avant-garde Research (X-star), Japan Agency for Marine-Earth Science and Technology (JAMSTEC), Yokosuka 237-0061, Japan

[2]Department of Biological Sciences, Hachioji Metropolitan University, Tokyo 192-0397, Japan

[3]Research Institute of Industrial Technology, Toyo University, Kawagoe 350-8585, Japan

*Correspondence

Shigeru Kawai: kawais@jamstec.go.jp

Yusuke Tsukatani: tsukatani@jamstec.go.jp

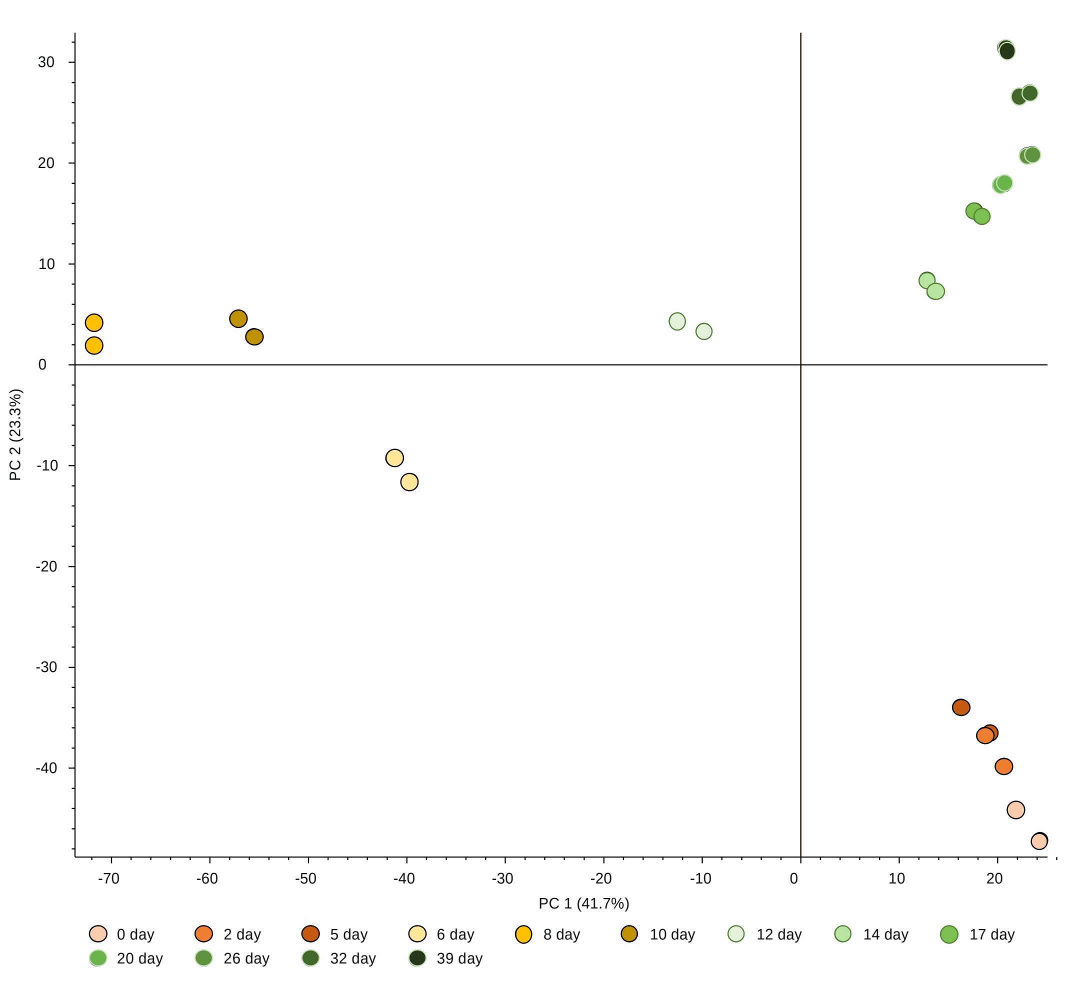

Supplementary Figure S1. Principal component analysis of proteomic datasets. Circles in the same color represent datasets from the same sampling date. Clustering of duplicate datasets in PCA indicates the two proteomic analyses are reproducible.

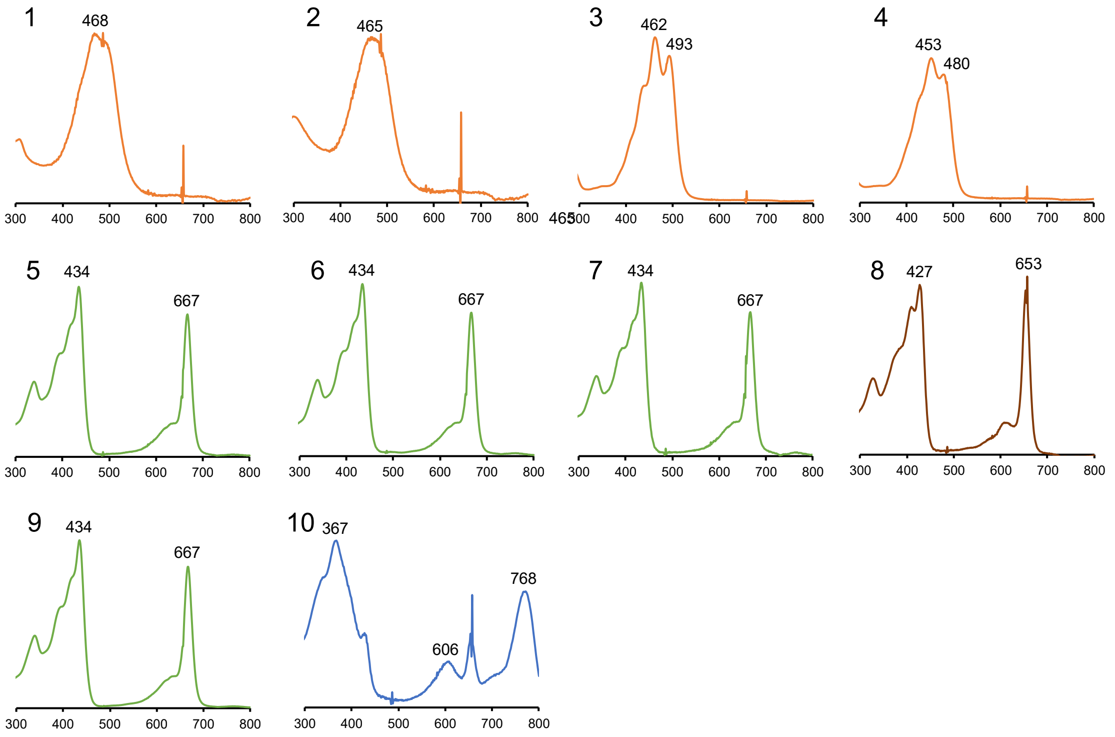

Supplementary Figure S2. Absorption spectra of peaks detected in HPLC analysis. Putative assignments of peaks are as follows: 1, 4-keto-γ-carotene; 2, echinenone; 3, γ-carotene; 4, β-carotene; 5, 6, 7, and 9, BChl *c*; 8, BChl *d*; 10, BChl *a*.
